# Important amino acid residues in the chloride pump halorhodopsin that accelerate ion transport despite no direct interaction with the substrate

**DOI:** 10.1016/j.jbc.2025.110703

**Published:** 2025-09-11

**Authors:** Yubo Zhai, Anna Shimosaka, Takashi Tsukamoto, Takashi Kikukawa

**Affiliations:** 1 Graduate School of Life Science, Hokkaido University, Sapporo, Japan; 2 Faculty of Advanced Life Science, Hokkaido University, Sapporo, Japan

**Keywords:** membrane transport, chloride transport, membrane protein, photobiology, rhodopsin, ion pump, retinal proteins

## Abstract

Ion-pump rhodopsins are widely distributed photoactive membrane proteins found in microorganisms. Their cytoplasmic (CP) regions are predominantly hydrophobic, inherently restricting substrate ion permeation. However, these rhodopsins can rapidly transport substrate ions *via* photo-induced conformational changes. The well-characterized H^+^-pumping rhodopsins employ dissociable residues such as Asp, Glu, or Lys to mediate rapid H^+^ relay reactions along a transiently hydrated CP pathway. In contrast, the corresponding mechanisms in other ion pumps remain poorly understood. Here, we investigated the key factors contributing to ion transport by halorhodopsin (HR), a Cl^-^ pump from the archaeon *Natronomonas pharaonis* (NpHR). Upon photoactivation, NpHR creates a hydrated Cl^-^ transport pathway in its CP region, which is surrounded by bulky hydrophobic residues that do not directly interact with Cl^-^. However, mutations in specific hydrophobic residues significantly slow Cl^-^ transport. Notably, Phe211 and Leu214, located near the pathway exit, play critical roles. Mutations in these residues likely disrupt the proper positioning of the Lys215 sidechain, which inadvertently binds Cl^-^ from the surrounding solution and positions it in a way that obstructs Cl^-^ transport. As a result, ion passage is hindered, leading to the accumulation of long-lived intermediates. These findings suggest that the hydrophobic residues surrounding the pathway are not merely structural components. Instead, they are critical for enabling specific conformational changes that facilitate the formation of a hydrated channel, allowing efficient Cl^-^ conduction without obstruction.

Ion pumps play vital roles in cells by acting as energy converters. By utilizing external energy sources such as ATP, sunlight, or redox reactions, they actively transport substrate ions across membranes. The resulting electrochemical ion gradients drive various biochemical processes while also posing the risk of ionic backflow. Many ion pumps achieve unidirectional transport without ion leakage, indicating the presence of highly specialized mechanisms to overcome these challenges. In this study, we investigated the mechanism of a light-driven Cl^-^ pump protein, halorhodopsin (HR) (1, 2). The cytoplasmic (CP) side of HR is predominantly hydrophobic. To facilitate Cl^-^ transport in this region, HR appears to create a transiently hydrated pathway without relying on residues that directly interact with Cl^-^ (3). Our experimental results support this view but also suggest a more complex mechanism. Replacing specific hydrophobic residues significantly slow Cl^-^ transport, indicating that these residues play a strategic role in shaping the hydration pathway to enable efficient Cl^-^ conduction.

Rhodopsins are ubiquitous light-responsive membrane proteins (4, 5, 6, 7). In animals, rhodopsins primarily act as light sensors. In contrast, microbial rhodopsins exhibit a much wider range of functions, including serving as light-driven ion pumps, light-gated ion channels, and even light-activated enzymes. Among these microbial rhodopsins, light-driven ion pumps represent the largest functional class. They can be further categorized into outward H^+^ and Na^+^ pumps, as well as inward Cl^-^ and H^+^ pumps. Halorhodopsin (HR), the focus of this study, was the first Cl^-^ pump identified, originating from the highly halophilic archaeon *Halobacterium salinarum* (1, 2). Subsequently, several other Cl^-^ pump groups have been discovered in eubacteria from diverse environments (8, 9, 10, 11), suggesting that Cl^-^ pumps are widely distributed among microorganisms. In this study, we investigated the Cl^-^ translocation mechanism of HR, using the representative homolog from *Natronomonas pharaonis*, hereafter referred to as NpHR. NpHR is the best-characterized Cl^-^ pump rhodopsin to date, providing a valuable model for understanding the functional mechanisms of this protein class.

Most microbial rhodopsins contain the chromophore retinal in the all-*trans* configuration in their dark states. Upon photoexcitation, the retina isomerizes to the 13-*cis* form. This energized state of the protein thermally relaxes back to the original state through several structural intermediates. During this cyclic reaction, known as the photocycle, microbial rhodopsins perform their respective functions. The retinal binds to a specific Lys residue *via* a protonated Schiff base, located near the center of the transmembrane region (Fig. 1*A*, overall structure of NpHR). In ion-pumping rhodopsins, this centrally located Schiff base divides the ion transport pathway into two half-channels: the extracellular (EC) and cytoplasmic (CP) sides. Regardless of the type of transported ion, the EC half-channels are typically composed of hydrophilic residues and are rich in water molecules. In contrast, the CP half-channels are predominantly hydrophobic, containing numerous hydrophobic residues. This strong hydrophobicity is advantageous for preventing unfavorable ion leaks in the dark state, ensuring the integrity of the ion gradients. However, once activated, ion pumps overcome this hydrophobic barrier to rapidly transport substrate ions. This dual functionality highlights the necessity for "special mechanisms" that enable efficient ion transfer even within hydrophobic CP channels.

**Figure 1:**
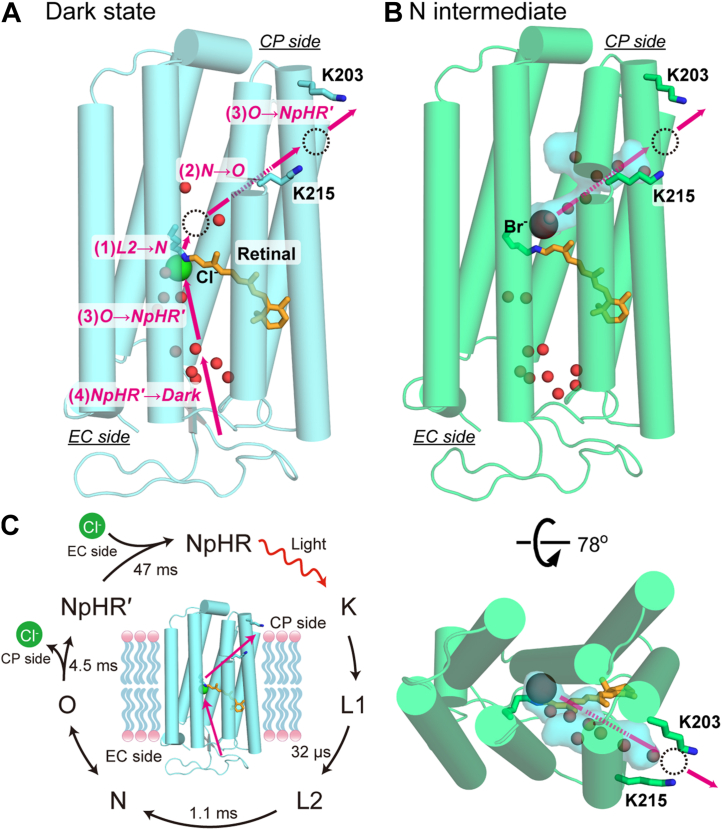
**Overview of the NpHR structure and Cl^-^ transfer reactions during the photocycle.** The Cl^-^ transfer timings are based on experimental results obtained at lower Cl^-^ concentrations (<100 mM) (20). *A*, tertiary structure of NpHR in the dark state (PDB ID: 3A7K). *Pink arrows* indicate the chloride transport steps. *Red* spheres and dotted circles represent water molecules and chloride binding sites at intermediates, respectively (3, 20). The exact position of the Cl^-^ binding site near Lys203 and Lys215 remains unidentified. At higher Cl^-^ concentrations (>100 mM), Cl^-^ may be released into the CP medium during the N → O transition. During the O → NpHR' transition, a new Cl^-^ is likely supplied to the original position, possibly from a site in the EC channel, although its precise location remains unknown (20). *B*, tertiary structure of NpHR in the N intermediate (PDB ID: 4QRY), where Br^-^ is used as a substrate ion instead of Cl^-^. The hydrated cavity that forms on the CP side is represented by a marine-colored surface, indicating the inflow of water molecules. *C*, photocycle scheme of NpHR. The lifetimes of the intermediates are shown based on previous experiments conducted at 1 M Cl^-^ in native NpHR-containing cell membranes (23). CP, cytoplasmic; NpHR, *Natronomonas pharaonis* halorhodopsin.

For H^+^-pumping rhodopsins, the special mechanism has been well studied, and proton "donor" residues are known to play essential roles (see reviews in (12, 13)). In photolyzed H^+^ pumps, the initial H^+^ transfer occurs from the protonated Schiff base to a H^+^ acceptor Asp residue in the EC channel. At a later stage in the photocycle, a new H^+^ is commonly supplied to the deprotonated Schiff base from the medium through the CP channel. This reaction is significantly accelerated by a conserved proton donor residue, which is located in the middle of the CP channel and is typically an Asp, Glu, or Lys residue (14, 15, 16, 17). Their removal greatly slows down the reprotonation of the Schiff base, effectively disabling H^+^ pumping activity under constant illumination (17). Similar to H^+^-pumping rhodopsins, HR can rapidly transport Cl^-^ through the CP channel. However, the special mechanism underlying HR's Cl^-^ transport remains poorly understood.

Unphotolyzed NpHR already binds the substrate Cl^-^, as shown in Figure 1*A* (18, 19). This Cl^-^ is translocated to the CP-side surface along the diagonal pathway, indicated by a pink arrow labeled " (2) N→O." This pathway was initially proposed based on the structure of the N intermediate (Fig. 1*B*), where the substrate ion (Br^-^, instead of Cl^-^) is located above the protonated Schiff base, and a hydrated channel appears diagonally. Our recent analyses further supported this diagonal pathway (20). In that study, we detected the release of Cl^-^ into the medium *via* a Cl^-^ binding site on the protein surface between Lys203 and Lys215. These residues are also shown in Figure 1, *A* and *B*.

As mentioned below, the hydrated channel is surrounded by numerous hydrophobic residues. Thus, NpHR appears to lack residues that are specifically arranged for Cl^-^ transport. Despite this seemingly simple architecture, Cl^-^ can traverse the channel at a rate comparable to the H^+^ transfer reactions of H^+^ pumps. In this study, we first explored the effects of replacing hydrophobic residues with weakly hydrophobic alanine. We observed that substitutions of Phe211 and Leu214 significantly slowed the Cl^-^ transfer reactions. These substitutions caused distortions in the photocycles, which persisted regardless of whether the replacements were with hydrophobic or hydrophilic residues. However, the distortions were largely mitigated by the simultaneous replacement of the neighboring Lys215 with a neutral amino acid. Thus, the substitution of Phe211 and Leu214 likely induces an unfavorable conformational change, which causes the neighboring Lys215 sidechain to inadvertently bind Cl^-^ from the surrounding solution and position it along the Cl^-^ transport pathway. Although the sidechains of Phe211 and Leu214 do not directly interact with Cl^-^, they appear to be critical for the rapid Cl^-^ transfer reaction.

## Results

### Probing the important residues surrounding the hydrated channel

Figure 1*C* illustrates the photo-intermediates that appear during the photocycle of NpHR. As each intermediate forms, Cl^-^ is translocated stepwise within the protein. These steps are indicated by pink arrows in the dark-state structure shown in Figure 1*A*. As mentioned earlier, upon the formation of the N intermediate, Cl^-^ moves to a site just above the Schiff base. At the same time, its exit pathway is created, forming a diagonal cavity that subsequently becomes hydrated (Fig. 1*B*).

**Figure 2:**
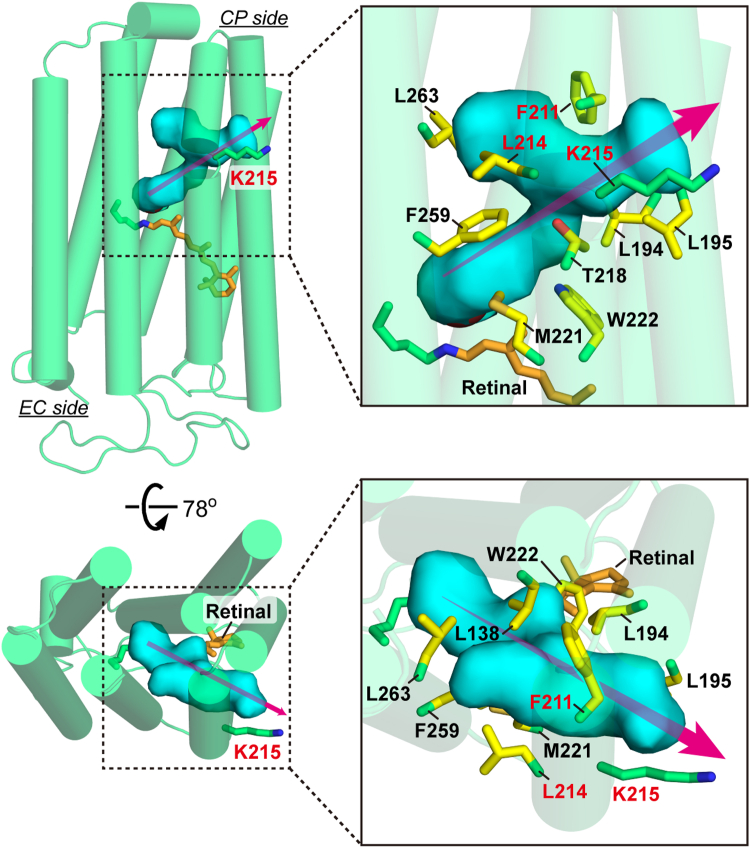
**Residues surrounding the hydrated channel in the N intermediate (PDB ID:****4QRY****).** The hydrated cavity is shown as a marine-colored surface and is surrounded by ten residues highlighted in *yellow*. The Lys215 residue, located on the protein surface rather than directly surrounding the channel, is shown in *green*. The key residues examined in this study are Phe211, Leu214, and Lys215, with their names highlighted in *red*.

**Figure 3:**
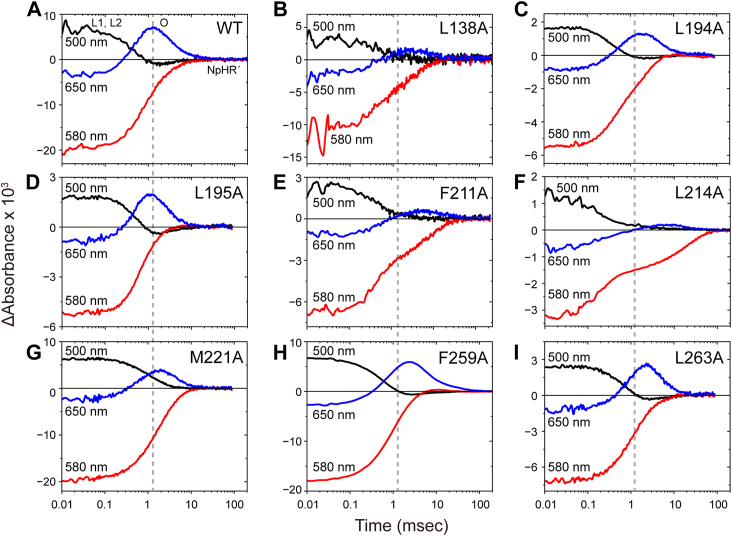
**Flash-induced absorbance changes of wild-type NpHR and eight mutants.***PanelA* represents the data for wild-type NpHR, whereas *panelsB–I* represent the data for the single-replacement mutants. Three colored lines represent absorbance changes at different wavelengths, reflecting the formation and decay of major intermediates. Each trace represents the average of 30 to 100 recordings of flash-induced absorbance changes. *Gray* dashed vertical lines indicate the time points of maximum O intermediate accumulation in wild-type NpHR. The buffer conditions were 10 mM MOPS (pH 7.0) containing 100 mM NaCl and 1.0% DDM. DDM, n-dodecyl-β-D-maltopyranoside; NpHR, *Natronomonas pharaonis* halorhodopsin.

Figure 2 provides an enlarged view of the hydrated region, highlighting 10 sidechains (colored in yellow) that surround the hydrated channel. Additionally, the sidechain of Lys215 (colored in green), which is not involved in surrounding the pathway, is also shown. The 10 residues around the pathway are highly conserved among HRs (Fig. S1). Among them, only Thr218 and Trp222 are hydrophilic or partially hydrophilic, while the remaining sidechains are bulky and highly hydrophobic. In a previous study, we generated various mutants of Thr218 (21). Replacement with hydrophobic residues resulted in a greater accumulation of the N intermediate, suggesting stronger Cl^-^ trapping at its binding site. However, this effect was not critical, as the overall photocycle rate remained largely unchanged. Therefore, in this study, we focused on the other nine residues and uniformly replaced them with weakly hydrophobic alanine. The resulting effects were examined using flash-induced absorbance changes during the photocycle. The results at 100 mM Cl^-^ are summarized in Figure 3, where data for the W222A mutant are absent due to unsuccessful expression in *Escherichia coli*. These experiments were conducted using *E. coli* lysates, which resulted in relatively large noise in some datasets due to lower NpHR expression levels. Each panel in Figure 3 presents time-traces of absorbance changes at three selected wavelengths.

In the dark state, NpHR exhibits an absorption maximum around 580 nm. Thus, negative deflections at this wavelength correspond to depletion and subsequent recovery of the original dark state. In contrast, at 500 nm, the traces initially show positive values and then decay, indicating the accumulation of the L1 and the subsequent L2 intermediates. The formation of L1 is extremely rapid, completing within 10 μs. Therefore, our data reflect the processes occurring after the formation of L1. As the 500 nm signal decays, a positive peak appears at 650 nm, corresponding to the formation of the O intermediate. The preceding N and O intermediates exist in a quasi-equilibrium state (Fig. 1*C*) (22, 23). For wild-type NpHR, this equilibrium is largely shifted toward O at 100 mM Cl^-^. The N intermediate has almost the same absorption spectrum as L1 and L2, and its accumulation in wild-type NpHR is minimal (Fig. 3*A*). Consequently, at 100 mM Cl^-^, the 500 nm signal decays completely when O accumulation reaches its maximum. As next NpHR' intermediate exhibits an absorption spectrum nearly identical to the dark state (22, 23), the 650 nm signal decreases while the 580 nm signal increases, ultimately returning to its initial level.

The alanine substitutions affected the photocycles to varying degrees. In Figure 3, vertical lines indicate the time point at which O accumulation reaches its maximum in wild-type NpHR. Mutants L194A, L195A, M221A, F259A, and L263A (Fig. 3, *C*, *D*, *G*, *H*, and *I*) exhibited relatively minor effects. While O accumulation was slightly reduced in some cases, overall photocycle progression remained largely intact. In contrast, O accumulation was significantly diminished in L138A, F211A, and L214A mutants (Fig. 3, *B*, *E*, and *F*). Notably, F211A and L214A also exhibited significantly slower kinetics. In particular, L214A showed a remarkably slow recovery of the original state (580 nm trace). Thus, the most distorted photocycles were observed in L214A, followed by F211A.

In both mutants, the 500 nm traces decayed to zero when the 650 nm traces reached their peak values. This suggests that, as in wild-type NpHR, the N intermediate did not accumulate significantly at this stage. Nevertheless, the positive peaks at 650 nm were notably small, indicating that the observed signal may not represent O intermediate accumulation. Instead, an unknown intermediate, with a λ_max_ distinct from 500 nm and 650 nm, likely accumulates in these mutants. Therefore, substitutions of Phe211 and Leu214 appear to significantly affect the formation of O, a key process in Cl^-^ translocation along the CP channel (Fig. 1*A*).

### Further effects of Phe211 and Leu214 substitutions

**Figure 4:**
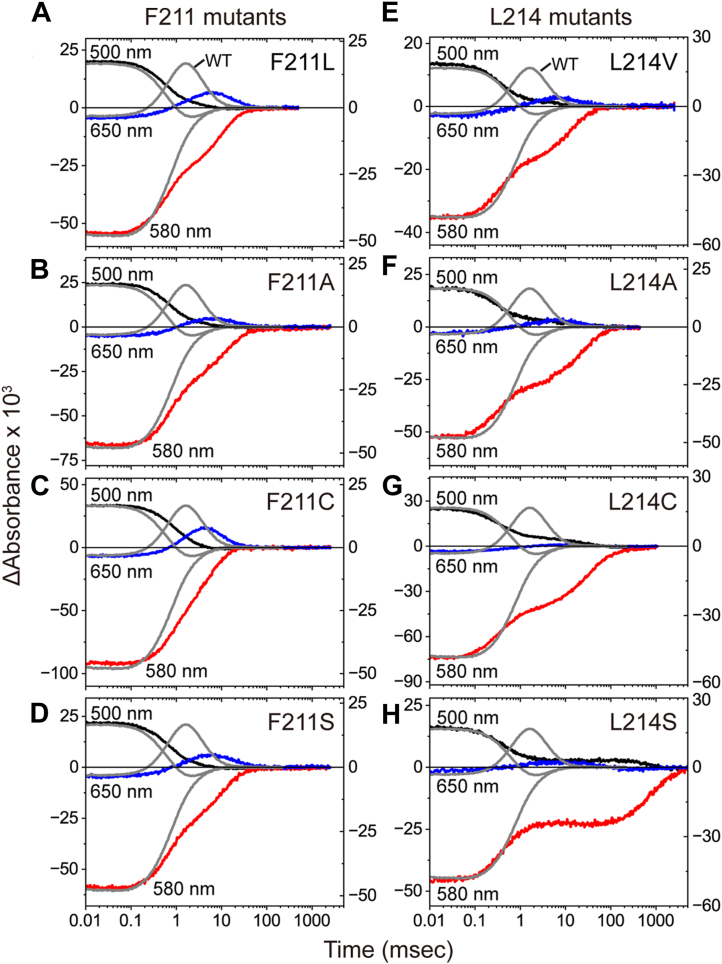
**Flash-induced absorbance changes of Phe211 and Leu214 mutants.***PanelsA–D* show the data for Phe211 mutants, whereas *panelsE–H* show the data for L214 mutants. Three colored lines represent absorbance changes at different wavelengths and are plotted on the left axes. Gray lines in all panels represent the absorbance changes of wild-type NpHR for comparison and are plotted on the right axes. Each trace represents the average of 30 recordings of flash-induced absorbance changes. The buffer conditions were 10 mM MOPS (pH 7.0) containing 100 mM NaCl and 0.05% DDM. The time traces of wild-type NpHR are shown in both Figures 8 and S4*A*, while those of F211A, F211S, L214C, and L214S appear in Figures 7, *A*–*D* and S4, *B*–*E*, respectively. DDM, n-dodecyl-β-D-maltopyranoside; NpHR, *Natronomonas pharaonis* halorhodopsin.

To further confirm the importance of these residues, we replaced them with three additional amino acids. All samples were purified from *E. coli* membranes and analyzed using flash-photolysis experiments (Fig. 4). The data for purified alanine mutants (F211A and L214A) are also included (Fig. 4, *B* and *F*).

In Figure 4, panels are arranged according to the hydrophobicity of the introduced side chains. Each panel also includes data for wild-type NpHR (gray lines). For both positions 211 and 214, replacement with smaller and more hydrophilic residues caused more severe distortions in the photocycles. The most striking effect was observed in the L214S mutant (Fig. 4*H*), which required several seconds to complete the photocycle. Such an unusually slow photocycle has never been reported in NpHR mutants. Other mutations in Figure 4 had smaller effects compared to L214S. However, even replacements with similar sidechains, such as F211L and L214V (Fig. 4, *A* and *E*), produced distinct changes in the latter half of the photocycle. In all mutants, the 650-nm peak was substantially reduced, and its timing was noticeably delayed. These findings suggest that Phe211 and Leu214 are irreplaceable for maintaining a rapid photoreaction.

Besides Cl^-^, NpHR also transports larger anions such as Br^-^, I^-^, and NO_3_^-^ (24, 25). Fig. S2 shows the effects of Phe211 and Leu214 mutations on the photoreactions during Br^-^ and NO_3_^-^ transport. In both cases, all mutants exhibited substantial distortions in the photoreactions, essentially identical to those observed during Cl^-^ transport. These findings indicate that Phe211 and Leu214 are important for anion transport regardless of the type of anion.

The absorption spectra of unphotolyzed NpHR provide further insight into these mutation effects. Fig. S3 compares the absorption spectra of wild-type NpHR and the Phe211 and Leu214 mutants. As shown, all spectra maintain nearly identical shapes with a λ_max_ of approximately 580 nm. Thus, these mutations did not induce obvious structural changes in the dark state. Instead, their effects became prominent during the photocycle, supporting the hypothesis that Phe211 and Leu214 play a crucial role in facilitating rapid Cl^-^ translocation.

### Reduced Cl^-^ pumping activities of the Phe211 and Leu214 mutants

A slower photocycle should result in a smaller amount of Cl^-^ transported under constant illumination. This assumption was experimentally confirmed using suspensions of *E. coli* cells expressing NpHR. Inward Cl^-^ transport by NpHR generates a negative membrane potential, which in turn drives passive H^+^ inflow in the presence of a protonophore. The resulting pH increase was detected by an electrochemical cell equipped with two indium–tin oxide (ITO) electrodes, functioning as a fast-response pH electrode (26). In *E. coli* suspensions, this pH increase could be detected as a voltage difference between the two electrodes during just 1 s of illumination (for details, see Experimental Procedures).

**Figure 5:**
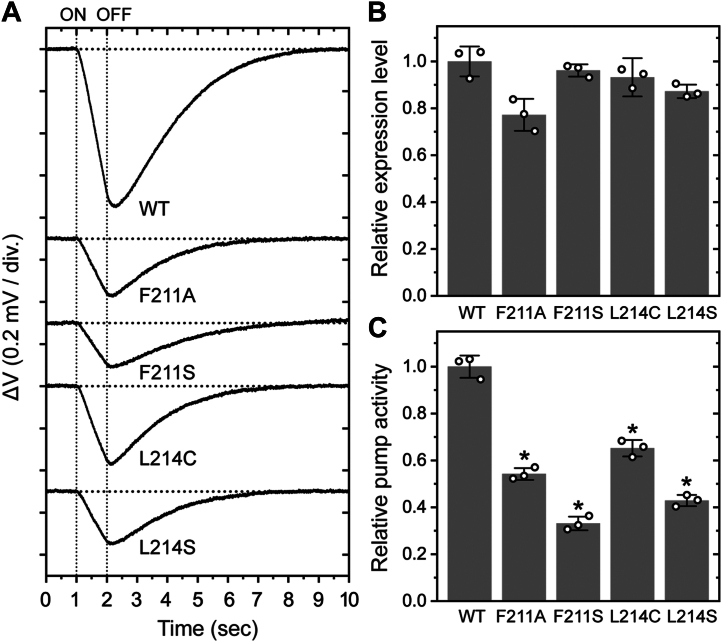
**Cl^-^-pump activities of wild-type NpHR and the mutants.***A*, time courses of light-induced voltage changes recorded by ITO electrodes in the electrochemical cell. *E. coli* cells were suspended in 300 mM NaCl containing 10 μM CCCP. Actinic light was applied for 1 to 2 s. For each sample, the voltage change was measured 9 to 10 times, and the averaged traces were used for subsequent analysis. Three independent experiments were performed (n = 3). Representative averaged traces are shown. *B*, relative expression levels in *E. coli* membranes. After transport measurements, *E. coli* cells were collected and lysates were prepared. The expression level of each protein was evaluated from the maximum value of the flash-induced absorbance change at 580 nm. Relative values are plotted as open circles. Bars represent the mean, and error bars indicate ±SD (n = 3). No statistical analysis was performed for expression levels. *C*, relative Cl^-^-pump activities. The slopes of the light-induced voltage changes were determined by fitting analysis for each averaged trace (n = 3). These slopes were normalized to the corresponding protein expression levels of each sample, and the resulting relative values are plotted as open circles. Bars represent the mean, and error bars indicate ±SD. Statistical significance was assessed by one-way analysis of variance (ANOVA), followed by Dunnett’s multiple comparisons test to evaluate differences between each mutant and wild-type NpHR (F(4,10) = 179.8, *p* < 0.0001). Asterisks (∗) indicate a statistically significant difference from wild-type NpHR (adjusted *p* < 0.0001 for all mutants; Dunnett’s test). CCCP, carbonyl cyanide *m*-chlorophenylhydrazone; NpHR, *Natronomonas pharaonis* halorhodopsin; SD, standard deviation.

The detected light-induced voltage changes are summarized in Figure 5*A*. A negative voltage change corresponds to a pH increase in the *E. coli* suspensions. To improve the signal-to-noise ratio, measurements were averaged over 9 to 10 trials for each sample. Compared with wild-type NpHR, all mutants exhibited smaller pH changes. To evaluate the relative pump activities, the slopes of the voltage changes were determined by linear fitting and then normalized to the relative amounts of expressed protein (Fig. 5*B*), which were independently estimated from flash-induced absorbance changes (for details, see Experimental Procedures). As shown in Figure 5*C*, all mutants exhibited substantially reduced pumping activities, to 30 to 65% of the wild-type level.

### Detailed analysis of distorted photocycles

As mentioned earlier, the photocycles of the Phe211 and Leu214 mutants appear to involve an intermediate distinct from the O intermediate of wild-type NpHR. To investigate this further, we performed a detailed analysis of the photocycles of the F211A, F211S, L214C, and L214S mutants, as these exhibited the most distorted photocycles among the mutants examined (Fig. 4). We conducted a global fitting analysis on datasets measured at 32 wavelengths (400–710 nm, at 10 nm intervals) using the sequential irreversible model (27), which describes the photocycle as a series of one-way transitions: P_0_→P_1_→P_2_→⋅⋅⋅→P_n_→P_0_. Here, P_0_ represents the original dark state, while P_i_ (i = 1, 2, … n) denotes kinetically distinguishable states that do not necessarily correspond to true physical intermediates. If multiple intermediates are connected by reversible reactions, they may coexist within the same P_i_ state. The number of P states (n) corresponds to the number of exponents used in the multiexponential fitting function. For wild-type NpHR, four exponents are sufficient to describe the photocycle (22, 23). However, for the mutants, we varied the number of exponents to determine the optimal n value, ultimately adopting six exponents for L214S and five for the other mutants. Fig. S4 summarizes the fitting results, including the overlaid time traces of the measured flash-induced absorbance changes with their best-fit curves, the decay time constants of the Pi states with standard errors, and the calculated time courses of changes in Pi-state concentrations.

**Figure 6:**
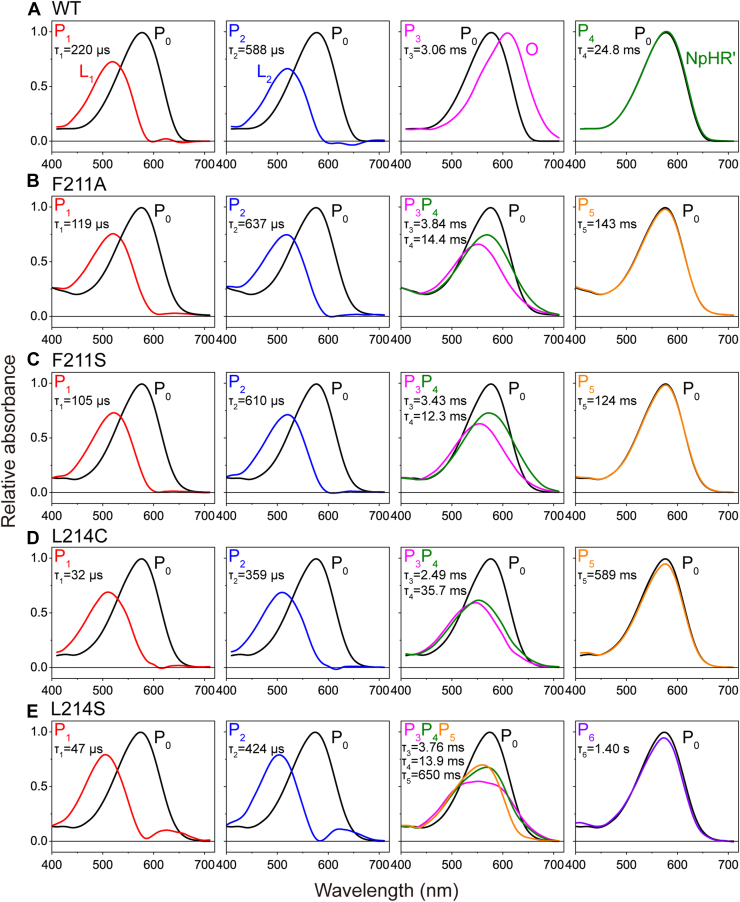
**Global fitting analysis of the photocycles of wild-type NpHR and the Phe211 and Leu214 mutants.** The *panelsA–E* show the data for wild-type NpHR, F211A, F211S, L214C, and L214S, respectively. The absolute spectra of the Pi states, obtained from global fitting analysis of 32 wavelengths (400–710 nm, 10 nm intervals), are shown along with their decay time constants. Data at each wavelength represent the average of 30 recordings of flash-induced absorbance changes. Further details of the fitting results are provided in Fig. S4, which shows overlaid traces of the measured data with the best-fit curves at three representative wavelengths, decay time constants of Pi states with fitting errors, and the time courses of Pi-state concentration changes. The P_0_ spectrum of wild-type NpHR (A) is also shown in Fig. S7 (*blue line*). NpHR, *Natronomonas pharaonis* halorhodopsin.

Using the determined fitting parameters, we calculated the absorption spectra of the P states to identify the physical intermediates involved. The results are summarized in Figure 6, where four panels illustrate the respective P state spectra. The decay time constants for each P state, denoted as τ_i_ (i = 1–n), are also shown.

For all samples, the first and second panels (left two columns) correspond to P_1_ and P_2_, which contain single intermediates with a λ_max_ around 510 nm. These are assigned to the L1 and L2 intermediates, indicating that the mutations do not significantly alter the process up to L2 formation, where Cl^-^ remains near its original position (Fig. 1*A*) (3). Similarly, the fourth panels (right-most column) contain single intermediates with spectra nearly identical to the dark state. These are assigned to NpHR', the final intermediate where a new Cl^-^ has already bound near the protonated Schiff base.

In contrast, the third panels exhibit the most significant mutation effects. For wild-type NpHR, this panel contains only the P_3_ spectrum, which corresponds to the O intermediate. At high Cl^-^ concentrations, the preceding N intermediate can also appear in P_3_ due to a quasi-equilibrium between N and O (22, 23). However, this equilibrium is strongly biased toward O at 100 mM Cl^-^. The N intermediate has a similar spectrum to L2, while O exhibits a redshifted spectrum, indicating the absence of Cl^-^ inside the protein. In our previous study, we confirmed that Cl^-^ is transported to the protein surface between Lys203 and Lys215 during O formation (20). Thus, in wild-type NpHR, Cl^-^ translocation is completed by the time O (P_3_) is formed, with a time constant of τ_2_ = 588 μs.

Notably, the O intermediate is absent in the third panels of all mutants. Instead, these panels contain two or three P states (P_3_ and P_4_, or P_3_, P_4_, and P_5_), all of which share broad spectra with λ_max_ around 540 to 550 nm. These spectra suggest that these states may contain mixtures of intermediates with different λ_max_ values. Their exact assignments remain unclear, but it is evident that these panels do not contain well-characterized intermediates such as L2, N, or O. We refer to these unidentified intermediates collectively as "X" intermediates. The λ_max_ of X is close to that of the dark state, suggesting that Cl^-^ remains trapped inside the protein and continues to influence the retinal's electronic state.

As mentioned earlier, Cl^-^ transfer through the CP channel occurs in wild-type NpHR with a time constant of τ_2_ = 588 μs. When does this transfer occur in the mutants? Since Cl^-^ remains bound in the X intermediates, its transfer likely coincides with the slowest decay of these states. Compared to wild-type NpHR, the slowest time constants of X are significantly prolonged: F211A, 14.4 ms (24 × slower); F211S, 12.3 ms (20 × slower); L214C, 35.7 ms (61 × slower); and L214S, 650 ms (307 × slower). Thus, all mutants lose the ability to rapidly transport Cl^-^ through the CP channel. Furthermore, the right-most panels, which primarily contain NpHR', also exhibit prolonged decay times, indicating that these mutations delay the final recovery from NpHR' to the dark state. This effect is addressed in the Discussion section.

### Unfavorable participation of Lys215 in Cl^-^ transfer

The absorption spectra of the X intermediates suggest that Cl^-^ remains near the binding site in the N intermediate. However, Phe211 and Leu214 are positioned near the protein surface. Why, then, do their mutations lead to the formation of X intermediates? One possible explanation involves Lys215, a positively charged residue located near Phe211 and Leu214. A plausible hypothesis is that these mutations displace the Lys215 sidechain, creating an unfavorable Cl^-^ binding site. If this binding site is positioned near the exit of the Cl^-^ transport pathway and has already bound Cl^-^ from the surrounding solution, the Cl^-^ migrating through the pathway from inside the protein may be unable to reach the exit.

**Figure 7:**
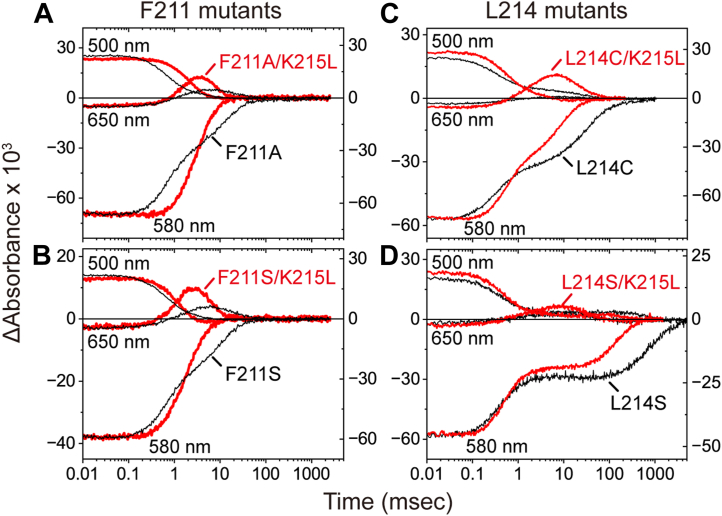
**Flash-induced absorbance changes of F211/K215 and L214/K215 double mutants.***PanelsA* and *B* show the data for F211 mutants, whereas *panelsC* and *D* show the data for L214 mutants. The absorbance changes of the double mutants are shown in *red* and plotted on the *left* axes. The *black* lines represent data for the respective F211 and L214 single mutants and are plotted on the *right* axes. Each trace represents the average of 30 recordings of flash-induced absorbance changes. The buffer conditions were 10 mM MOPS (pH 7.0) containing 0.1 M NaCl and 0.05% DDM. The time traces of F211A, F211S, L214C, and L214S are identical to those in Figures 4, *B*, *D*, *G*, *H* and S4, *B*–*E*, respectively. DDM, n-dodecyl-β-D-maltopyranoside.

To test this hypothesis, we constructed double mutants in which Lys215 was replaced with a neutral leucine in addition to the mutations at either Phe211 or Leu214. The effects of these mutations on flash-induced absorbance changes are shown in Figure 7 for Cl^-^ transport and in Fig. S5 for Br^-^ and NO_3_^-^ transport. Compared to the single mutants (black lines), the double mutants (red lines) exhibited substantially improved photocycle kinetics, regardless of the anion type. For the Phe211 mutants (left panels of Figs. 7 and S5), both double mutants showed photocycles closely resembling those of wild-type NpHR (grey traces in Figs. 4 and S2). For the Leu214 double mutants (right panels of Figs. 7 and S5), significant improvements were also observed, although some distortions remained, particularly in the L214S mutants. In these double mutants, the serine and cysteine side chains may form a weak anion-binding site through interactions with nearby hydrophilic components.

**Figure 8:**
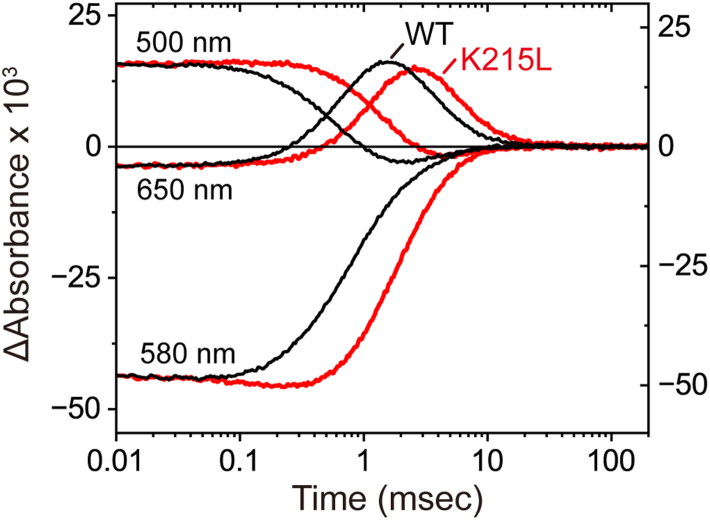
**Comparison of the photocycles of wild-type NpHR and the K215L single mutant.** Each trace represents the average of 30 recordings of flash-induced absorbance changes. The buffer conditions were 10 mM MOPS (pH 7.0) containing 0.1 M NaCl and 0.05% DDM. The data for the K215L mutant are plotted on the *left axis*, while those for wild-type NpHR are plotted on the *right axis*. The time traces of wild-type NpHR are identical to those in Figures 4 and S4*A*. DDM, n-dodecyl-β-D-maltopyranoside; NpHR, *Natronomonas pharaonis* halorhodopsin.

These findings indicate that in the single Phe211 and Leu214 mutants, Lys215 adopts an unfavorable position within the Cl^-^ transport pathway, impeding efficient ion transfer. Lys215 is highly conserved among HRs (Fig. S1), suggesting it plays a critical role in Cl^-^ transport. To further investigate this role, we examined the photocycle kinetics of the K215L single mutant (Fig. 8). This mutant exhibited a slower photocycle, in which O formation was more strongly affected by the mutation than O decay. These results suggest that Lys215 facilitates rapid Cl^-^ translocation along the CP channel.

## Discussion

In this study, we investigated the Cl^-^ transfer reaction on the CP side of NpHR, which is predominantly composed of hydrophobic residues. During the formation of the N intermediate, large conformational changes occur in these residues, creating a diagonal cavity across the CP side that subsequently becomes hydrated to facilitate Cl^-^ movement. This hydrated channel is primarily surrounded by bulky hydrophobic residues that do not directly interact with Cl^-^. Consequently, these residues were initially thought to play only a minor role in Cl^-^ transfer. However, our mutational analyses revealed that replacing several of these hydrophobic residues with alanine caused distortions in the latter half of the photocycle, indicating their functional involvement in Cl^-^ transport. Among these residues, Phe211 and Leu214—both located near the channel exit—proved to be particularly critical, as their substitution resulted in the formation of the characteristic intermediates X.

### A model involving the participation of Lys215

As described earlier, in wild-type NpHR, Cl^-^ near the retinal migrates toward the CP surface in the vicinity of Lys215 during O formation (20), and is subsequently released into the medium during O decay. Through this transient Cl^-^ binding, the Lys215 residue may facilitate rapid Cl^-^ migration along the CP channel. This interpretation is consistent with the slower formation and decay of O observed in the K215L mutant (Fig. 8). In contrast to its positive role in the wild-type protein, Lys215 may play a negative role when the neighboring Phe211 or Leu214 is substituted. Upon such mutations, the Lys215 sidechain may shift into the Cl^-^ transport pathway, forming an unfavorable Cl^-^ binding site. If this site strongly binds Cl^-^ from the surrounding solution prior to O formation, the migrating Cl^-^ from inside the protein may be prevented from reaching the exit, ultimately blocking CP-channel transport. This scenario could account for the formation of long-lived X intermediates. Thus, Phe211 and Leu214 are likely important for enabling the conformational changes required to establish a well-structured hydration pathway, ensuring the unobstructed passage of Cl^-^.

A key assumption of this model is the formation of an unfavorable Cl^-^ binding site near Lys215 in the mutants. This possibility should be experimentally examined in future work. Because this putative binding site would be spatially distant from the retinal, the binding of Cl^-^ itself would likely have negligible effects on the retinal’s absorption spectrum. Instead, Cl^-^-induced conformational changes in the retinal and nearby protein moiety could potentially be detected using vibrational spectroscopic techniques such as Raman scattering or Fourier-transform infrared spectroscopy. Targeted experiments of this nature should be performed to test this hypothesis and to further clarify the role of Lys215 in CP-channel transport.

Beyond the formation of X intermediates, the decay of the final intermediate, NpHR', was also markedly slowed (right-most panels in Fig. 6). As previously noted, NpHR' formation is associated with Cl^-^ release into the CP medium (20). In the Phe211 and Leu214 mutants, this Cl^-^ release might be hindered, potentially leaving Cl^-^ bound at the CP surface of NpHR'. Such retention might substantially delay the restoration of the protein to its original dark state.

### Cl^-^ pumping activity under constant illumination

The functional importance of Phe211 and Leu214 was further supported by their Cl^-^ transport activities (Fig. 5). Consistent with the kinetic data, the mutants exhibited reduced pumping under constant illumination, where a faster photocycle would normally yield higher activity. The substantial slowing of the photocycle in the L214S mutant was therefore expected to cause a pronounced activity loss. The smaller-than-expected reduction might be explained by photoexcitation of long-lived X and NpHR' intermediates. If these states can either rapidly revert to the dark state or directly initiate the next Cl^-^ transport cycle upon illumination, the apparent reaction rate under constant light would increase. Such a “bypass” process has been proposed for NpHR' in wild-type NpHR (28).

### Tentative model for the consequences of Phe211 and Leu214 mutations

**Figure 9:**
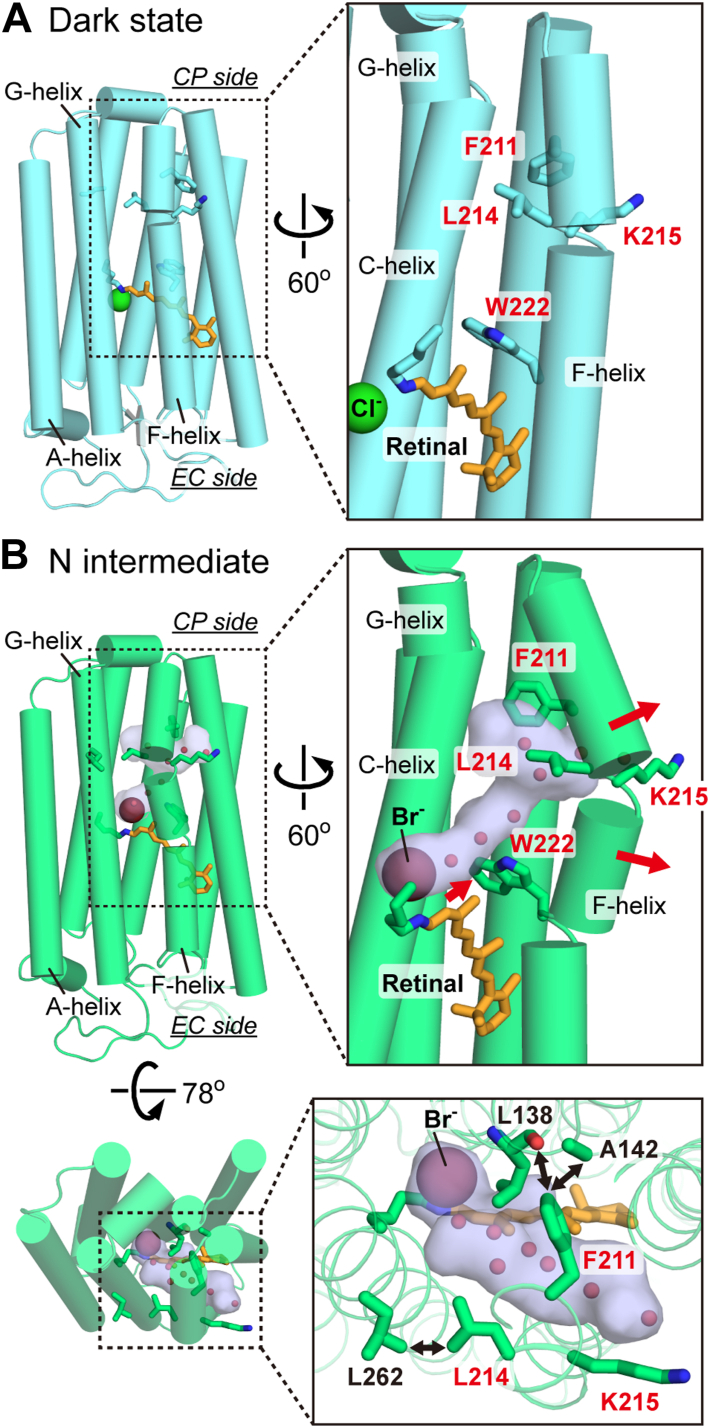
**Comparison of the dark state and N intermediate structures of NpHR.***A*, dark state structure. *B*, N intermediate structure. In the *top* and *middle right panels*, only the *upper* part of the G-helix is shown, while the rest of the G-helix is omitted for clarity. Other helices are displayed as usual. In the *bottom right panel*, possible steric hindrances around Phe211 and Leu214 in the N intermediate state are indicated by double-headed arrows. PDB IDs 3A7K and 4QRY correspond to the dark state and N intermediate, respectively. NpHR, *Natronomonas pharaonis* halorhodopsin.

In wild-type NpHR, the Lys215 sidechain projects outward from the protein, positioned too far from the CP channel to impede Cl^-^ passage. In the mutants, however, Lys215 is likely displaced into the transport pathway. In the N intermediate of wild-type NpHR, the CP half of the F-helix tilts outward (Fig. 9, *A* and *B*) due to steric repulsion between Trp222 and the retinal C13-methyl group, with the largest displacement occurring near Phe211, Leu214, and Lys215 (Fig. 9*B*). This tilt appears to be stabilized by local steric contacts—such as between Phe211 and Ala142 (C-helix), Phe211 and the carbonyl oxygen of Leu138 (C-helix), and Leu214 and Leu262 (G-helix). Mutations at Phe211 or Leu214 may weaken or disrupt these interactions, allowing Lys215 to intrude into the CP channel. This model is based solely on wild-type structural data; molecular dynamics simulations or related structural studies on the mutants will be required for verification.

### Comparison of the residues among Cl^-^ pump rhodopsins

This study demonstrates that hydrophobic residues not directly interacting with Cl^-^ can nonetheless play important roles in facilitating rapid Cl^-^ transfer along the CP channel. It is therefore intriguing to consider whether similar mechanisms are conserved in other Cl^-^ pumps. Fig. S1 compares the NpHR residues with those of representative cyanobacterial and marine eubacterial Cl^-^ pumps. The 11 residues listed on the left correspond to those surrounding the Cl^-^ transfer pathway in NpHR, as well as Lys215, which faces outward.

Cyanobacterial Cl^-^ pumps share a high degree of conservation with archaeal Cl^-^ pumps: of the 10 residues surrounding the pathway, seven are conserved, including Phe211 and Leu214 of NpHR. In the Cl^-^ pump from *Synechocystis* sp. PCC7509 (SyHR), a diagonal Cl^-^ transport pathway similar to that of NpHR, has been suggested from its crystal structure (29), with water-filled cavities near His167, the counterpart of Thr218 in NpHR. The reported importance of His167 for SyHR anion pump activity (30) is consistent with this diagonal pathway. Although cyanobacterial Cl^-^ pumps retain a hydrophobic Val or Leu in place of Lys215, they possess two Arg residues at positions corresponding to residues 199 and 212 of NpHR. Fig. S6, *A* and *B* show the positions of several residues in NpHR and in the Cl^-^ pump from *Mastigocladopsis repens* (MrHR). In MrHR, the Arg side chains face outward near the location of Lys215 in NpHR, suggesting that, although the details differ, cyanobacterial Cl^-^ pumps may use a mechanism broadly similar to that of NpHR.

Marine eubacterial Cl^-^ pumps also possess hydrophobic residues at positions corresponding to those surrounding the Cl^-^ transfer pathway in NpHR. However, many of their residues differ from those in NpHR; moreover, even between the two marine eubacterial Cl^-^ pumps compared, the amino acid composition varies considerably. Similar to Lys215 in NpHR, the Cl^-^ pump from *Fulvimarina pelagi* (FR) positions positively charged sidechains toward the exterior (Fig. S6*C*). Nevertheless, the low sequence similarity suggests that marine eubacterial Cl^-^ pumps likely employ a mechanism distinct from that of NpHR.

The movement of ions through a hydrophobic protein interior presents a major challenge for ion-transport proteins, making it plausible that “special mechanisms” have evolved to accomplish this task. Investigating the similarities and differences in Cl^-^ transfer mechanisms across the various Cl^-^ pump rhodopsins, therefore represents an important avenue for future research.

## Experimental procedures

### Gene preparation

*E. coli* strain DH5α was used for DNA manipulation. Plasmids for expressing various mutants were constructed using a QuikChange site-directed mutagenesis kit (Agilent Technologies) based on the original pET-NpHR plasmid (31). All plasmids encoded C-terminally histidine-tagged NpHR.

### Protein expression and purification for spectroscopic measurements

NpHR and its mutants were expressed and purified from *E. coli* BL21(DE3) cells as previously reported (32). Briefly, the cells were grown at 37 °C in 2 × YT medium supplemented with 50 μg/ml ampicillin. When the OD_600_ reached approximately 1.2 (AP-120, APEL), protein expression was induced by adding 1 mM isopropyl-β-D-thiogalactopyranoside in the presence of 10 μM all-*trans* retinal. After 3 h of induction, the cells were harvested by centrifugation (6700× *g*, 6 min at 4 °C), washed once with buffer (50 mM Tris-HCl, pH 8.0) containing 300 mM NaCl, and stored at −30 °C until use. The cells were resuspended and disrupted by sonication. The resulting membrane fractions were collected by ultracentrifugation (225,000× *g*, 60 min at 4 °C) and solubilized in the same buffer containing 1 M NaCl and 1.5% n-dodecyl-β-D-maltopyranoside (DDM) at 4 °C overnight. The solubilized NpHR proteins were purified using nickel-nitrilotriacetic acid-agarose. The purified samples were dialyzed against an appropriate buffer (10 mM MOPS, pH 7.0) containing 0.1 M NaCl and 0.05% DDM for spectral measurements.

### Absorption spectra and flash photolysis measurements

UV-visible spectra of NpHR samples were recorded at room temperature using a UV-1800 spectrometer (Shimadzu). Flash-induced absorbance changes were measured using a computer-controlled single-wavelength kinetics system, as previously described (33). The actinic light was the second harmonic of a Q-switched Nd: YAG laser (532 nm, 5 ns, and 2.0 mJ). At each measuring wavelength, 30 laser pulses were applied to improve the signal-to-noise ratio. Sample temperature was maintained at 20 °C using a circulating water bath. For measurements using purified proteins, NpHR concentration was adjusted to 10 to 15 μM, determined using an extinction coefficient of 54,000 M^−1^ cm^−1^ at 580 nm.

For initial measurements, we used *E. coli* lysates prepared as follows: NpHR-expressing cells were harvested, washed twice with 10 mM MOPS (pH 7.0) containing 100 mM NaCl, and resuspended in the same buffer to an OD_660_ of approximately 10.0. The suspensions were sonicated in the presence of 1.0% DDM, and the resulting lysates were used for flash-photolysis measurements.

### Cl^-^ pumping activity measurements

The activities of the wild-type NpHR, and its mutants were evaluated using *E. coli* C43(DE3) cells expressing the respective proteins. Cell suspensions were prepared as described previously (26). Briefly, after 4 h of protein expression induction, the cells were harvested by centrifugation and washed twice with 0.3 M NaCl. The pellet was resuspended in the same solution and incubated with gentle shaking at 4 °C for 40 h in the presence of 10 μM carbonyl cyanide m-chlorophenylhydrazone (CCCP). The cells were then washed twice with the same solution, and the optical density at 660 nm (OD_660_) was adjusted to 10.0.

Inward Cl^-^ transport generates negative membrane potential, which in turn drives H^+^ influx from the bulk medium, leading to a pH increase in the *E. coli* suspension. This pH change was measured using a custom-made electrochemical cell (26) consisting of two ITO electrodes and a dialysis membrane separating the *E. coli* suspension from the counter aqueous solution. Each ITO electrode was in contact with one of the two solutions, both containing 10 μM CCCP. The ITO electrodes detected pH changes, while illumination induced a pH increase only in the *E. coli* suspension, producing a voltage difference between the electrodes. This voltage difference was recorded. Owing to the rapid response of the ITO electrodes, a 1 s illumination was sufficient to detect Cl^-^ pump activity. To improve the signal-to-noise ratio, voltage traces for each sample were averaged over 9 to 10 trials. Illumination was provided by a 150 W xenon arc lamp combined with two glass filters (IRA-25S and Y-50; Toshiba). The light intensity at 530 nm was 28.4 mW cm^−2^, measured using an Orion-PD optical power meter (Ophir Optonics Ltd). All measurements were performed at room temperature (∼25 °C).

Anion transport activities were quantified as the slopes of the light-induced voltage changes, obtained by linear fitting of each average trace. Because these slopes depend on the expression level of the protein in *E. coli* cell membranes, expression levels were assessed from the amplitudes of flash-induced absorbance changes at 580 nm, corresponding to the maximum absorption of the dark state. After transport activity measurements, cells were pelleted by centrifugation, resuspended in 10 mM MOPS-NaOH buffer (pH 7.0) containing 0.1 M NaCl, and adjusted to OD_660_ = 10.0. Following the addition of 1% DDM, the cells were lysed by sonication. The resulting lysates were used to record flash-induced absorbance changes, with 30 traces averaged per sample. The maximum negative deflection (0.01–0.02 ms, 31 data points) was extracted, and the averaged value was used as a proxy for protein expression level. To account for expression differences among samples, transport activity (slope of voltage change) was normalized to the corresponding expression level.

### Global fitting analysis

Flash-induced absorbance changes collected at 400 to 710 nm (10 nm intervals) were analyzed using an irreversible sequential model (27), which represents the photocycle as a series of unidirectional reactions among kinetically distinguishable states P_i_ (i = 1, 2, … n): P_0_ → P_1_ → P_2_ → ⋯ → P_n_ → P_0_, where P_0_ corresponds to the dark state. This model assumes only forward reactions. Thus, P_i_ states do not necessarily correspond to discrete physical intermediates. If some intermediates are linked by fast reverse reactions, they may appear together within the same P_i_ state. The steps for this analysis have been described previously (34, 35). The noise level at each measuring wavelength was first determined by calculating the standard deviation (SD) of the data in the −40 to 0 ms time range. The reciprocals of these SD values were then used as weights for calculating the fitting residuals at each wavelength. Under optimal fitting conditions, the SD of the residuals becomes one at any wavelength. The data set comprising all wavelengths was then simultaneously fitted using multiexponential functions. After fitting, the difference spectra between P_i_ and P_0_ (Δ*ε*_i_) were calculated from the fitted parameters. The absolute absorption spectra of each P_i_ state were obtained by adding these difference spectra to the corrected dark-state spectrum (P_0_), which was obtained by subtracting the Rayleigh scattering component from the measured dark-state spectrum (see Fig. S7).

## Data availability

All of the data supporting the findings of this study are available within the paper and the supporting information.

## Supporting information

This article contains supporting information.

## Conflict of interest

The authors declare that they have no conflicts of interest with the contents of this article.
