# Supplementary material for: Important amino acid residues in the chloride pump halorhodopsin that accelerate ion transport despite no direct interaction with the substrate
Source: J Biol Chem. 2025 Sep 11;301(10):110703. doi: 10.1016/j.jbc.2025.110703 (PMC12538430; doi:10.1016/j.jbc.2025.110703)
Supplement: Supporting Figures [file mmc1.pdf]

Supporting Information

Important amino acid residues in the chloride pump halorhodopsin that accelerate ion transport despite no direct interaction with the substrate

Yubo Zhai [1], Anna Shimosaka [1], Takashi Tsukamoto[1,2], Takashi Kikukawa[*,1,2]

[1]Graduate School of Life Science, Hokkaido University, Sapporo, 060-0810, Japan

[2]Faculty of Advanced Life Science, Hokkaido University, Sapporo, 060-0810, Japan

[*] Corresponding author

Dr. Takashi Kikukawa, E-mail: kikukawa@sci.hokudai.ac.jp.

Table of Contents

Figure S1. Comparison of residues among archaeal HRs and eubacterial Cl^- pump rhodopsins.............S2

Figure S2. Flash-induced absorbance changes of the Phe211 and Leu214 mutants during Br^- and NO_3^- transport reactions..S3

Figure S3. Absorption spectra of wild-type NpHR and Phe211 and Leu214 replacement mutants…........S4

Figure S4. Global fitting results of the flash-induced absorbance changes of wild-type NpHR and the Phe211 and Leu214 mutants...S5

Figure S5. Flash-induced absorbance changes of the F211/K215 and L214/K215 double mutants associated with Br^- and NO_3^- transport reactions...S7

Figure S6. Comparison of the residues of focus in this study on the tertiary structures of NpHR, MrHR, and FR...S8

Figure S7. Determination of the P_0 spectrum...S9

			10 residues surrounding the CP channel and Lys215 facing outward												
	Species	Accession ID	138	194	195	211	214	215	218	221	222	259	263	199	212
Archaeal Cl^- pump (halorhodopsin)	*Natronomonas pharaonis* (NpHR)	CAI48412	L	L	L	F	L	K	T	M	W	F	L	A	S
	Halobacterium salinarum	CAP13054	L	L	V	F	L	R	T	L	W	F	L	A	D
	Halobacterium salinarum shark	BAA07822	L	L	L	F	L	K	T	L	W	F	L	P	G
	Halobacterium salinarum port	Q48315	L	L	L	F	L	K	T	L	W	F	L	P	G
	Haloarcula vallismortis	BAA06679	L	L	L	F	L	K	T	L	W	F	L	P	G
	Halobacterium sp. SG1	CAA49773	V	L	L	F	L	K	T	L	W	F	L	A	N
	Haloterrigena sp. Arg-4	BAA75201	L	L	L	F	L	R	T	L	W	F	L	P	G
	Halorubrum sodomense	BAA75202	L	L	L	F	L	K	T	L	W	F	L	A	N
Cyanobacterial Cl^- pump	*Mastigocladopsis repens* (MrHR)	WP_017314391	L	L	A	F	L	V	H	L	W	F	S	R	R
	Synechocystis sp. PCC 7509 (SyHR)	WP_009632765	L	L	V	F	L	L	H	L	W	F	S	R	R
Marine eubacterial Cl^- pump	*Nonlabens marinus* S1-08^{T} (NM-R3)	WP_052476770	L	V	G	I	V	F	M	A	W	Y	I	F	T
	Fulvimarina pelagi (FR)	WP_007065598	L	V	R	P	I	W	F	F	W	Y	L	I	K
	Residue numbers	MrHR	86	140	141	159	162	163	166	169	170	207	211	145	160
		SyHR	86	141	142	160	163	164	167	170	171	208	212	146	161
		NM-R3	110	171	172	190	193	194	197	200	201	238	242	176	191
		FR	122	183	184	202	205	206	209	212	213	250	254	187	203

Figure S1. Comparison of residues among archaeal HRs and eubacterial Cl^- pump rhodopsins. Numbers in the top row indicate the residue positions in NpHR. The background colors—grey, blue, and green—denote residues that are hydrophobic, highly basic (Lys, Arg), or have other features, respectively. Eleven residues located between positions 138 and 263 of NpHR are highly conserved among HRs. Of these, 10 residues, excluding Lys215, surround the diagonal Cl^- transport pathway. Residues that differ from the conserved ones among HRs are highlighted in red. The residues at positions 199 and 212 were not mutated in this study and are less conserved; thus, they are shown in blue. The cyanobacterial and marine eubacterial Cl^- pumps do not conserve the residue corresponding to Lys215 in NpHR, which faces the outside of the protein. However, the boxed residues in these pumps face the respective exterior and are located near the position equivalent to Lys215 in NpHR. The positions of these residues are indicated for MrHR and FR in their respective tertiary structures in Fig. S6. HR, halorhodopsin; NpHR, *Natronomonas pharaonis* HR; MrHR, *Mastigocladopsis repens* HR; FR, Cl^- pump rhodopsin from *Fulvimarina pelagi*.

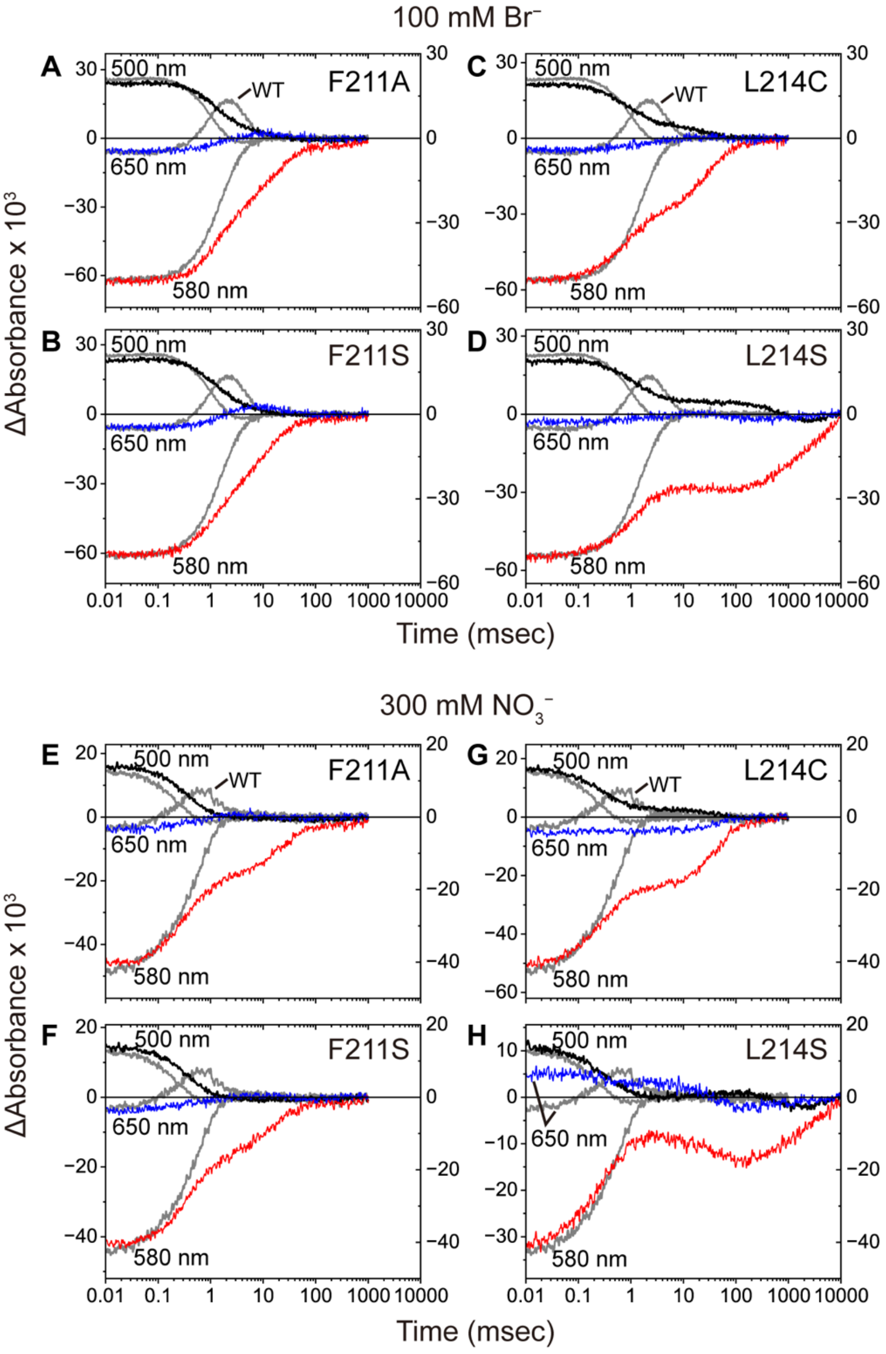

Figure S2. Flash-induced absorbance changes of the Phe211 and Leu214 mutants during Br^- and NO_3^- transport reactions. Three colored lines represent absorbance changes at different wavelengths and are plotted on the left axes. Gray lines in all panels represent the absorbance changes of wild-type NpHR for comparison and are plotted on the right axes. The data at each wavelength represent the average of 30 time traces of the flash-induced absorbance changes. The buffer conditions were 10 mM MOPS (pH 7.0) containing 0.05% DDM and either 100 mM NaBr or 300 mM $NaNO_3$. The time traces of NpHR mutants

in Fig. S2A-H are also shown in Fig. S5A-H (black lines), respectively. NpHR, *Natronomonas pharaonis* halorhodopsin; DDM, n-dodecyl-β-D-maltopyranoside.

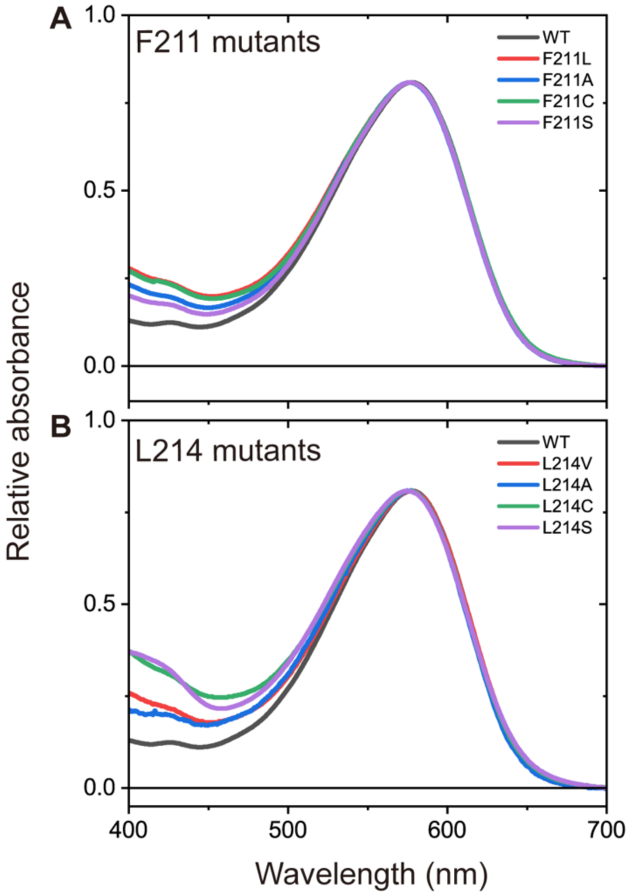

Figure S3. Absorption spectra of wild-type NpHR and Phe211 and Leu214 replacement mutants. The medium (pH 7.0) contained 10 mM MOPS, 0.1 M NaCl, and 0.05% DDM. The spectrum of wild-type NpHR is also shown in Fig. S7 (black line). NpHR, *Natronomonas pharaonis* halorhodopsin; DDM, n-dodecyl-β-D-maltopyranoside.

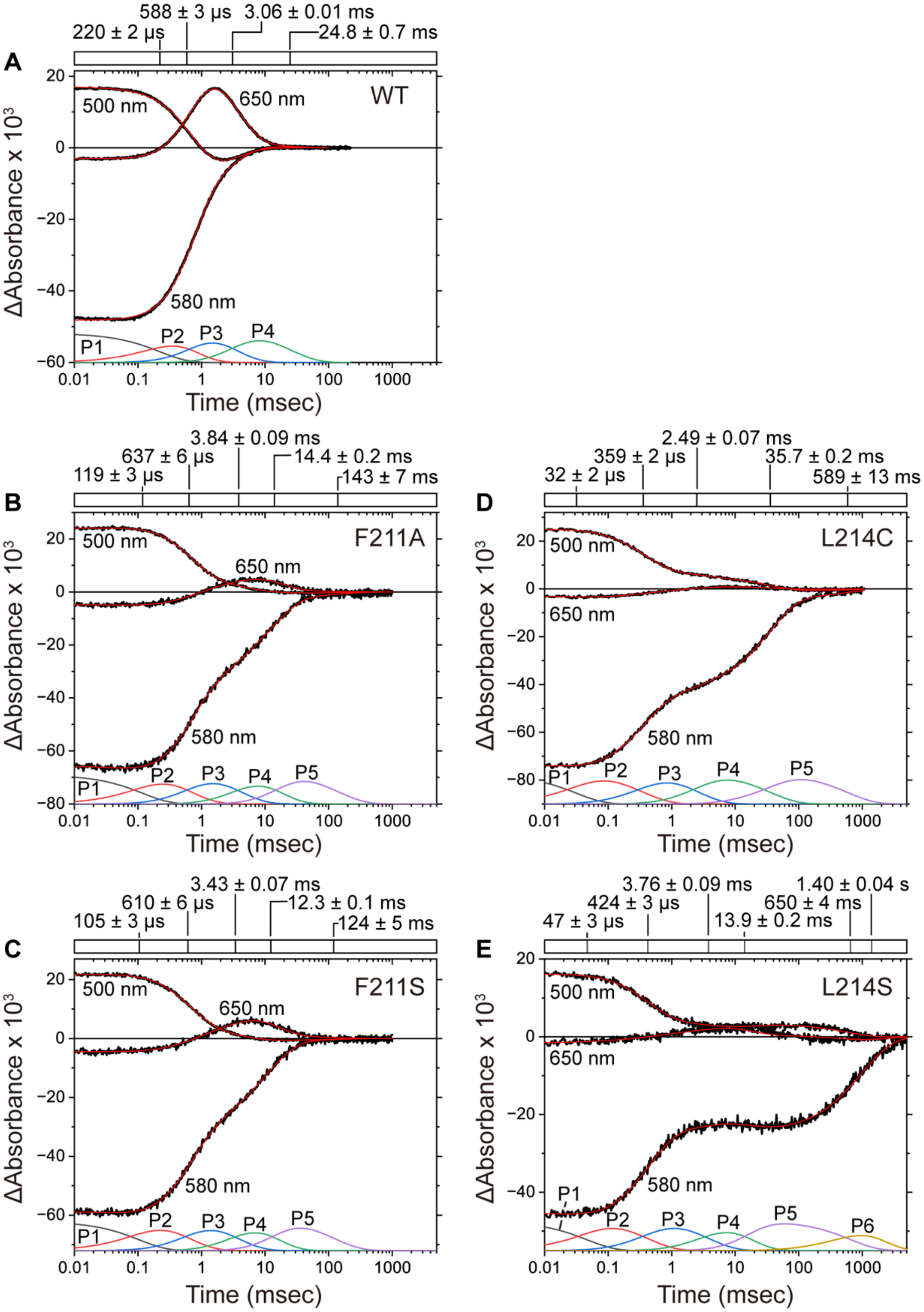

Figure S4. Global fitting results of the flash-induced absorbance changes of wild-type NpHR and the Phe211 and Leu214 mutants. The flash-induced absorbance changes measured at 32 wavelengths (400–710 nm, 10 nm intervals) were fitted simultaneously. Each dataset represents the average of 30 time traces.

The thin panels show the decay time constants of the Pi states, with errors indicating the standard errors of the fits. The large panels display the measured data (black lines) at three representative wavelengths, the corresponding fitting curves (red lines), and the calculated concentration changes of the Pi states, plotted with the bottom axis as the baseline. The measured data for wild-type NpHR (A) are identical to those in Fig. 4 and Fig. 8, whereas those for NpHR mutants (B-E) are identical to those in Fig. 4B, D, G, and H and Fig. 7A-D, respectively. NpHR, *Natronomonas pharaonis* halorhodopsin.

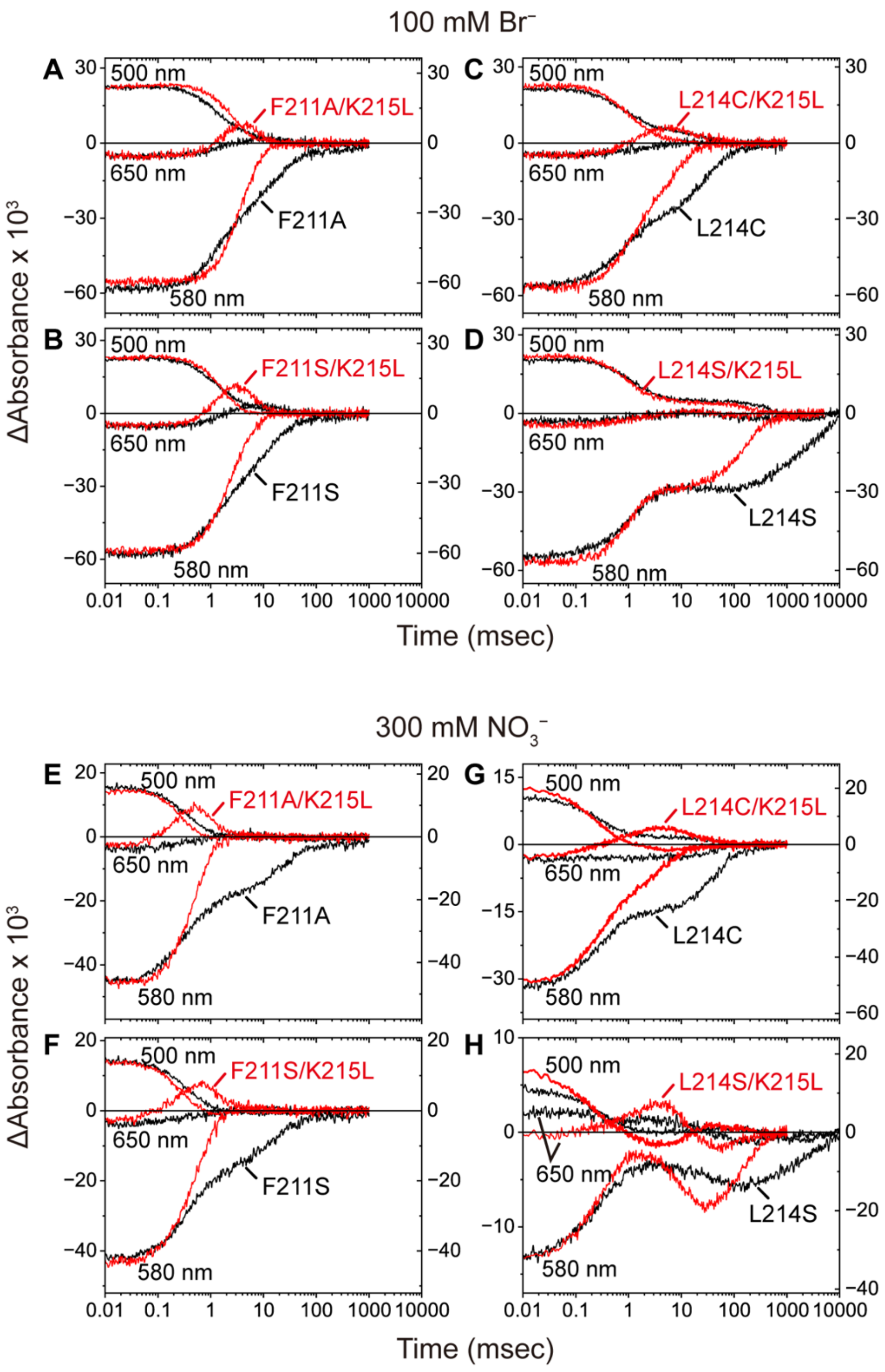

Figure S5. Flash-induced absorbance changes of the F211/K215 and L214/K215 double mutants associated with Br^- and NO_3^- transport reactions. The absorbance changes of the double mutants are shown in red and plotted on the left axes. The black lines represent data for the corresponding F211 and L214 single mutants and are plotted on the right axes. Each trace represents the average of 30 time traces of the flash-induced absorbance changes. The buffer conditions were 10 mM MOPS (pH 7.0) containing 0.05%

DDM and 0.1 M NaBr or 300 mM $NaNO_3$. The time traces of single mutants in Fig. S5A-H are also shown in Fig. S2A-H, respectively. DDM, n-dodecyl-β-D-maltopyranoside.

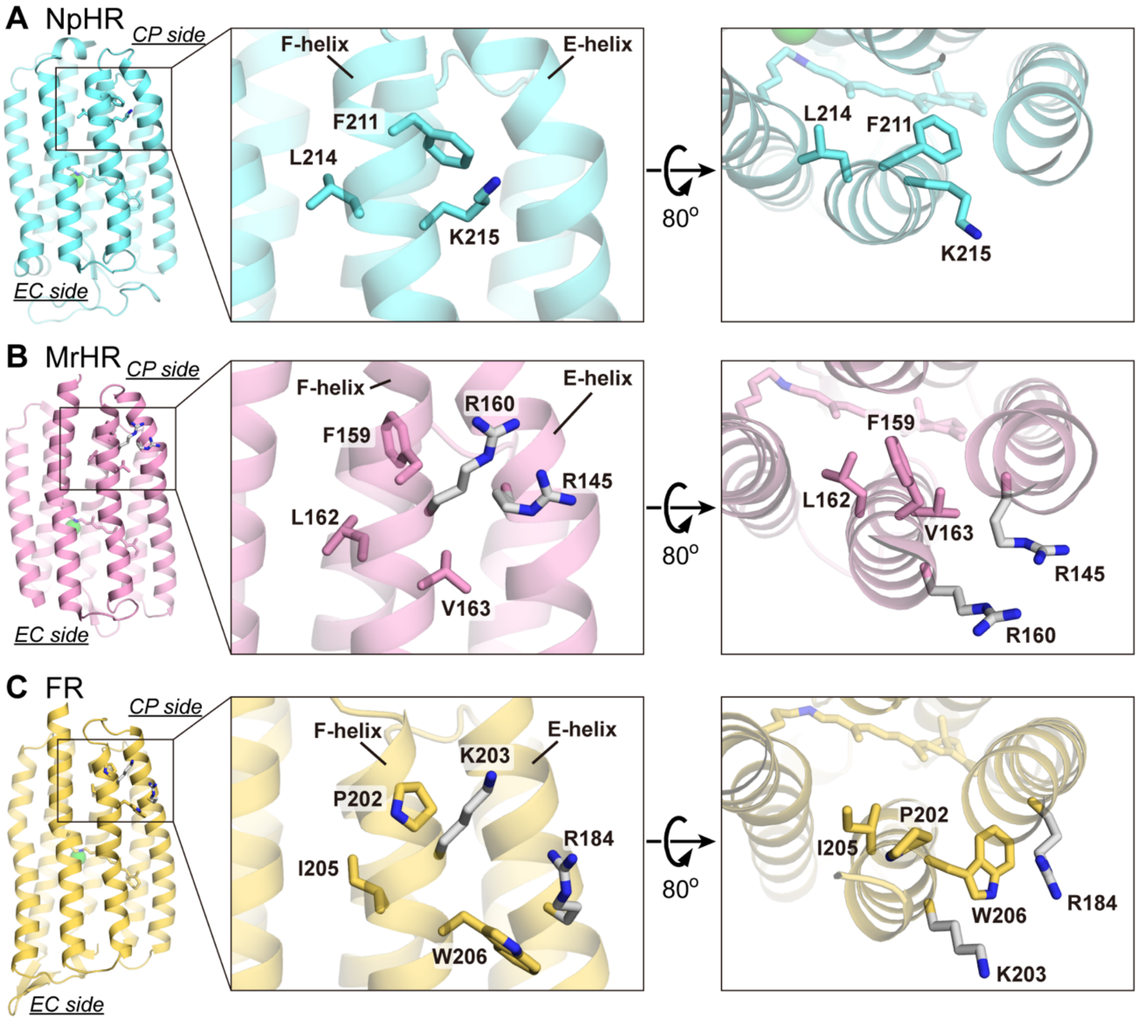

Figure S6. Comparison of the residues of focus in this study on the tertiary structures of NpHR, MrHR, and FR. Three residues corresponding to Phe211, Leu214, and Lys215 of NpHR (A) are shown in pink and yellow for MrHR (B) and FR (C), respectively. Neither MrHR nor FR conserves Lys215 of NpHR; however, both possess two positively charged residues facing the outside of the protein, similar to Lys215 of NpHR. These residues are shown in grey in panels (B) and (C). The PDB codes are 3A7K for NpHR and 6XL3 for MrHR. The structure of FR was generated by AlphaFold 3 (https://alphafoldserver.com). The predicted structure was very similar to the NM-R3 structure (PDB code: 5G28). NpHR,

Natronomonas pharaonis halorhodopsin; MrHR, *Mastigocladopsis repens* halorhodopsin; FR, Cl^- pump rhodopsin from *Fulvimarina pelagi*; NM-R3, Cl^- pump rhodopsin from *Nonlabens marinus* S1-08^T.

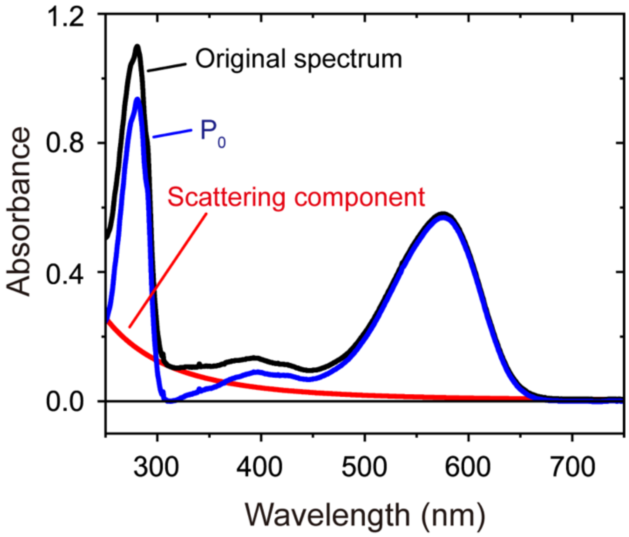

Figure S7. Determination of the P_0 spectrum. The procedure is shown only for wild-type NpHR. The scattering component (red line) was estimated using the α/λ^4 (λ in nm) term. This scattering curve was subtracted from the original spectrum (black line), and the remaining spectrum (blue line) was used as the P_0 spectrum. The original spectrum and P_0 spectrum are also shown in Fig. S3 and Fig. 6A (black lines), respectively. NpHR, *Natronomonas pharaonis* halorhodopsin.
